# Bottom-up microwave transformation of molecules to carbon dots for detection and encryption applications

**DOI:** 10.1039/d5ra08943c

**Published:** 2026-01-21

**Authors:** Arun Annamalai, Sundaravadivel Elumalai, Sambasivam Sangaraju, Fathy M. Hassan

**Affiliations:** a Department of Chemistry, United Arab Emirates University Al Ain 15551 United Arab Emirates f.hassan@uaeu.ac.ae; b National Water and Energy Center, United Arab Emirates University Al Ain 15551 United Arab Emirates s_sambasivam@uaeu.ac.ae; c HIDE- Laboratory, Department of Chemistry, Faculty of Engineering and Technology, SRM Institute of Science and Technology Kattankulathur Tamil Nadu 603203 India sundaravadivelchem@gmail.com

## Abstract

The development of low-cost, stable, and effective fluorescent materials for detecting carcinogenic water contaminants and antibiotic drugs is a significant step toward protecting the environment and public health. In this work, we have prepared CDs using simple precursors, tartaric acid and di-aminopropane, *via* a facile, fast, one-step microwave-assisted method in 4 minutes. The as-prepared CDs were thoroughly investigated using sophisticated analytical techniques, including UV-Vis, PL lifetime, HR-TEM, and XRD. The exciting fluorescent excitation-dependent and independent character was revealed by photoluminescence, XPS, and FT-IR measurements, and it was found that CDs were made with a uniform core and an electron-rich surface functional group. Also, the prepared CDs exhibit greater stability in various environmental conditions. Furthermore, the core fluorescent character of CDs was effectively employed to detect Cr^6+^ and doxycycline, with lower detection limits of 0.14 and 0.09 µM, among the various metal cation and antibiotic groups. Additionally, it retains its sensitivity in the presence of multiple co-existing metal cations and antibiotics individually. In addition to environmental protection, we have utilized CDs for the secure transport of information *via* fluorescent ink and anti-counterfeiting security features. This present work displays the multifunctional ability of CDs, that can serve as a potential sensor for toxic metal ions and antibiotics in water based environments and also an excellent information encryptor for secure information transportation.

## Abstract 2

Multifunctional carbon dots from tartaric acid and diaminopropane prepared using a microwave assisted method have the potential to serve as a dual contaminant sensor and invisible security ink for secure information transmission.

## Graphical abstract

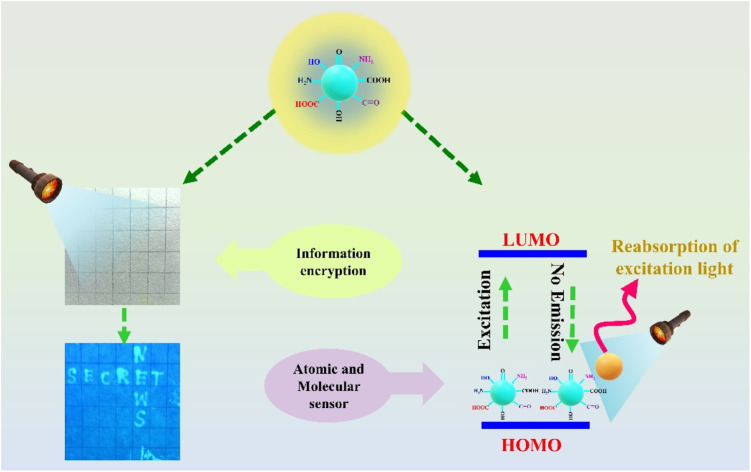

## 1 Introduction

Carbon dots (CDs) are the new and powerful 0D material in the kingdom of carbon-based nanomaterials. These CDs entice the entire research community with unique and superior characteristics, such as excitation-dependent emission, upconverted photoluminescence, extreme aqueous solubility, and nanoscale size, as well as comparatively lower toxicity, which underscore their exceptional properties compared to other quantum dots. These characteristics enable CDs to be employed in numerous research areas, including fluorescence sensors, supercapacitors, solar cells, anticancer agents, electrocatalysts, LEDs, photocatalysts, and drug delivery agents.^1^ Similarly, CDs can be prepared by multiple methods, including hydrothermal, microwave irradiation, solvothermal, ultrasonication, laser ablation, electrochemical, and direct pyrolysis. Among the vast array of synthetic approaches, microwave-assisted preparation of CDs is considered a straightforward, fast, safe, and economical method.^2^ However, despite being two decades old, the exact structure and origin of fluorescence remain under debate. Moreover, the reports state that CDs are constructed with a crystalline sp^2^-hybridised core, followed by shielding with functional groups.^3^

Regarding the origin of fluorescence, there are three possible mechanisms. Molecular surface defects, which arise from the effect of defects and *in situ* or *ex situ* doping during the preparation of CDs, are followed by the quantum confinement effect, where the emission depends on the size of CDs. The third one represents the core and surface state, meaning CDs resemble a core (carbon) and shell (functional group) type material; meanwhile, most research reports strongly support core and surface state mechanisms for the origin of fluorescence and multiple emissions from CDs.^4–7^ Also, a report from K.C. Lin's research group detailed and highlighted the multiple emissions was occurred from the core and surface structure and pointed out that choosing of precursor plays a important role in the structural and fluorescence emission behaviour of CDs, which confirms that the preparation of CDs from the very simple structured precursor allows us to predict the optical and structural characteristics of the CDs.^8^ One of the most pressing challenges facing the entire globe is the lack of access to clean water. Clean water is the primary source of life for humans, animals, and plants. Nowadays, water quality has deteriorated due to the massive release of pollutants from the dye, chemical, and pharmaceutical industries, posing significant threats to all living creatures. Therefore, our ecology became more polluted due to the accumulation of scum in the aquatic system. Early exposure to toxins in an aqueous system can reduce the risk of harm. Usually, hexavalent chromium Cr(vi) is a type of industrial pollutant released from leather, steel, and other chemical industries. Due to its higher water-soluble quality, it easily mixes with ground and surface water bodies.^9–11^

Contaminated water easily enters the human body through the food chain and can seriously affect organs such as the kidneys, liver, and lungs. Similarly, doxycycline is a typical antibiotic widely prescribed to treat respiratory problems, urinary tract infections and sinusitis. Apart from human medication, it is widely used as a feed additive in poultry and aquaculture farming, as a dietary supplement. The primary concern arises when the excessive doxycycline usage in livestock may mix with our dairy products, such as meat, eggs and milk, which directly enters the human body and causes acute damage to organs, especially cause damage to kidneys, liver, and hypertension and also causes allergies.^12–17^ Therefore, early detection of hexavalent chromium and doxycycline helps us avoid these concerns. Meanwhile, for the detection of toxins in aqueous conditions, numerous instrumental-based systems are available, including fluorescence (photoluminescence-PL), calorimetry, electrochemistry, high-performance liquid chromatography (HPLC), inductively coupled plasma optical emission spectroscopy (ICP-OES), and surface-enhanced Raman spectroscopy (SERS). While discussing the various detection processes, the fluorescence (PL)-based discrimination process is relatively simple compared to other detection methods.^18–21^ In particular, it took a few seconds to prepare the substrate, and the analysis of the sample also delivered results rapidly.

In this research, we planned and successfully prepared CDs from simple chemical precursors using an ultrafast microwave-assisted method, eliminating the need for additional solvents in a single-step process. Interestingly, the as-prepared CDs exhibit blue colour emission, and their characteristics are investigated using multiple sophisticated analytical techniques. From this, it was confirmed that the CDs exhibit greater water solubility, excitation-dependent emission with a well-defined average lifetime in the excited state, a well-functionalized structure with a molecular-state emission origin, and outstanding optical stability under various conditions. CDs selectively detect dual analytes, such as Cr^6+^ and Doxycycline, in aqueous environments and retain their selectivity even in the presence of various common substances. We then attempted to utilize the CDs' fluorescence as a fluorescent ink for the easy, secure transmission of important data and information. Our work identified a pathway for developing a multi-functional CD using a simple precursor and method.

## 2 Experimental section

### 2.1 Materials

1,2-diaminopropane was obtained from sigma aldrich, and tartaric acid, ammonium acetate, ammonium fluoride, ammonium iodide, ammonium phosphate, ammonium bromide, ammonium chloride, KOH, FeCl_3_·6H_2_O, CuCl_2_·2H_2_O, NiCl_2_·6H_2_O, MnCl_2_·4H_2_O, SnCl_4_·5H_2_O, MnCl_2_·4H_2_O, LiCl_2_, CaCl_2,_ Pb (NO_3_)_2_, Hg (CH_3_COO)_2_, CdCl_2_, NaCl, Zn (CH_3_COO)_2_·2H_2_O, CoCl_2_·6H_2_O, and K_2_Cr_2_O_7_ were purchased sisco research laboratories private limited. All the chemicals used here are not subjected to any additional purification.

### 2.2 Characterization techniques

The as-prepared CDs are studied using the following sophisticated instrumentation facilities. The optical absorption characteristics are recorded with the aid of a Shimadzu UV 3600 Plus, and the photoluminescent emission and average lifetime of CDs and CDs with analytes are noted using an Edinburgh Instruments (FLS 1000). The presence of prefunctional groups is analyzed using FTIR-ATR on a Shimadzu IR Tracer 100. Further investigation of the deep functional group was conducted using X-ray photoelectron spectroscopy (XPS) (ULVAC-PHI 5000 probe III XPS). The crystal phase of the CDs is analyzed using an X-ray diffractometer from PANalytical, Netherlands. TEM and HR-TEM images are captured with a high-resolution transmission electron microscope (JEOL JEM-2100 Plus HR-TEM). The surface electric charge was determined with the Malvern Panalytical.

### 2.3 Microwave-assisted preparation of CDs

**Fig. 1:**
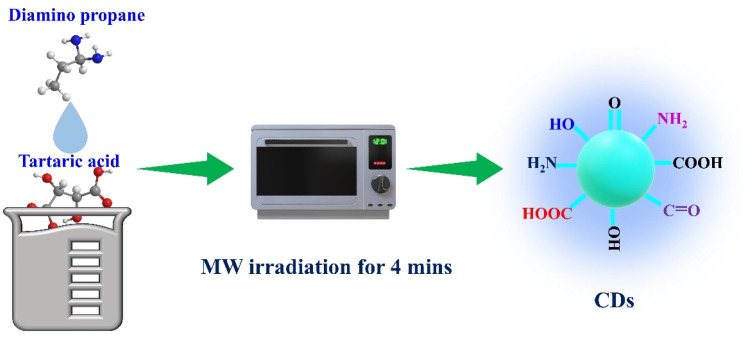
Microwave-assisted synthesis outline of CDs.

The preparation of blue-emitting CDs follows: 500 mg of tartaric acid (TA) was completely dissolved in 500 µL of water, followed by the addition of exactly 2 mL of diamino propane (DAP). The mixture was stirred until a homogeneous solution was obtained. After that, the entire reactant mixture was placed in a household microwave oven, and the heating parameters were set to 700 W; the reaction time was then set to 4 minutes. After 4 minutes, a dark brown, carbonized product precipitate was obtained, and it was then cooled to room temperature. After that, the dark brown precipitate dissolved in water and was purified using a 0.22 µm syringe filter. Finally, the obtained supernatant solution was stored at room temperature to carry out further analysis. The entire CDs preparation process is picturized in (Fig. 1).

### 2.4 Fluorescent detection of water toxins with CDs

For the detection of waterborne toxins, CDs were used as probes in a fluorescence-based method. In the detection setup, we prepared standard stock solutions (1 mM) of individual metal cations and pharmaceutical chemicals. After preparing the analyte and probe solution, the preparation proceeds in a quartz cuvette, where exactly 100 µL of probe (CDs) solution is dispersed in 2.5 mL of DI water (optimized concentration ratio based on fluorescence intensity). Metal cations and pharmaceutical chemicals with known concentrations were individually transferred into a newly prepared probe solution for CDs. Then, the prepared setup was transferred to a fluorescence instrument, where the screen emission character of the probe was assessed immediately after the addition of the analyte. The changes in the CDs emission intensity after the addition of analyte were noted at the maximum emission wavelength observed during optimization 446 nm, excited at 360 nm. Although the sensitivity of CDs analyzed in the presence of Cr^6+^ and Doxycycline, along with other metal cations, common anions, and pharmaceutical compounds.

## 3 Results and discussion

### 3.1 Optical properties of CDs

**Fig. 2:**
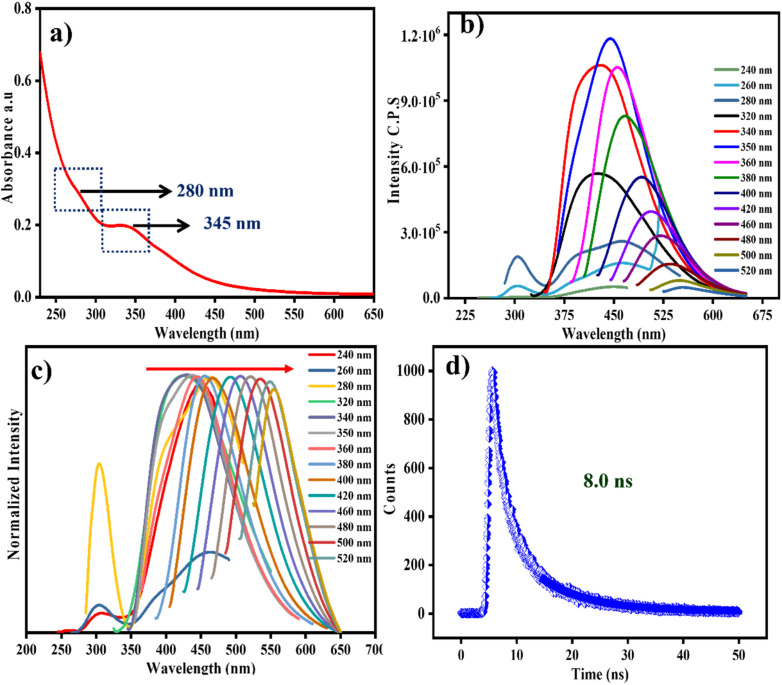
(a) UV-Vis absorption spectra, (b) emission spectrum recorded at different wavelengths of excitations, (c) excitation wavelength-dependent emission spectra of CDs, (d) lifetime spectra of CDs.

The results obtained after UV-Vis and photoluminescent spectra allow for an easy understanding of the optical characteristics of CDs. Two peaks were observed in the CD absorption spectra at 280 and 345 nm. The minimized hump at 280 nm, caused by a π–π* transition, is observed for an aromatic C=C moiety featuring sp^2^ hybridization, which may be present in the core structure of the CDs (Fig. 2a). Followed by a clear hump at 345 nm, attained due to the transition between *n*–π* energy levels, which confirms the occurrence of multiple functional groups on the CDs surface structure.^22^ One can notice that the peak at 280 nm appears significantly less intense than the peak at 345 nm (Fig. 2b). This may be due to the aromatic C=C structure being assembled as the core structure inside the CDs, while the functional groups are arranged on the surface of CDs. Therefore, the C=C moiety within the CDs cannot absorb more incident light than the functional moieties on the surface.^23^

The photoluminescence (PL) emission spectra was further verified to strengthen the structural properties of the CDs. In that, a weak emission spectrum was observed while exiting the CDs at 240, 260, and 280 nm, with corresponding emission maxima of 310 nm, indicating an excitation-independent emission process. On the other hand, the same CDs exhibit distinct excitation-wavelength-dependent emission properties (Fig. 2c), in which the emission peak gradually shifted towards longer wavelengths as the excitation wavelength increased from 320 to 520 nm. The excitation-dependent emission response indicates the presence of multiple functional groups on the surface of CDs, such as –NH3, –COOH, –OH, and C–N, which generate additional emissive states based on the different energy levels of the surface functional groups.^23^ However, the excitation independent emission is ascribed to the relatively uniform C=C moiety within the inner core of CDs. Additionally, the fluorescence intensity originating from the surface states of the CDs is higher than that from the core states. This difference in the emission intensity is likely due to the rapid recombination rate of excitons (electron–hole pairs) at the surface state compared its core recombination process.^8,24^ In addition, the time-resolved PL decay spectra (Fig. 2d) recorded with an excitation wavelength of 340 nm yield an average excited-state lifetime of 8.0 ns, further confirming that the recombination rate of surface-state CDs is greater.^25^

Meanwhile, the fluorescent materials must maintain their optical stability in the face of common environmental conditions. The interference of ionic species existences employing NaCl (from 0–200 mM) (Fig. S2a), followed by storage stability (prolonged storage period) was evaluated with an interval of 60 days (Fig. S2b), and photostability by constant irradiation of UV light (365 nm) for 60 minutes (Fig. S2c). However, the attained results show that the as-prepared CDs exhibit excellent stability in various environmental conditions. Nevertheless, the effect of fluorescent emission under different pH conditions (2–11) studied in an HCl-NaOH environment was entirely different (Fig. S2d). The bar graph shows that at acidic pH, the fluorescence intensity of CDs was reduced, and it increased with a gradual increase in pH towards alkaline. The interesting results indicate that CDs are structured with rich amine-based functional groups. At acidic pH, the amine-based moiety, such as –NH_2_, converts to NH^3+^ ions, making free localisation difficult; thereby, fluorescence emission is reduced. On the other hand, under an alkaline pH environment, the emission intensity of CDs increased due to the possibility of localisation of free electrons in the –NH_2_ moiety.

### 3.2 Structural and surface characterisation

**Fig. 3:**
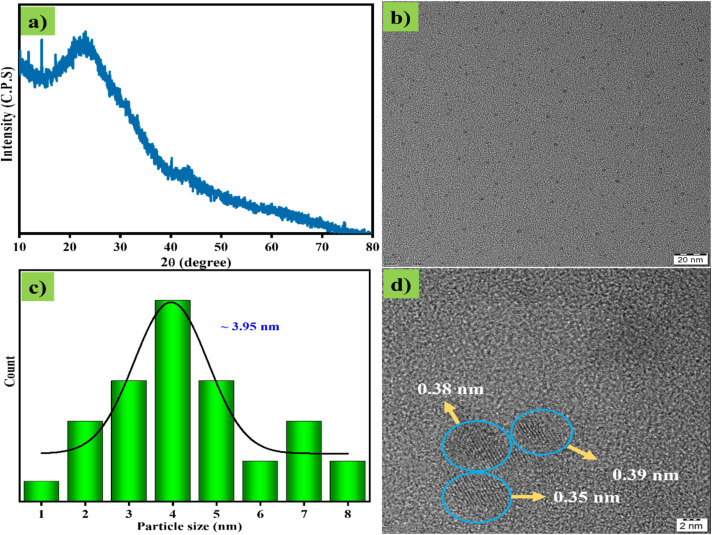
(a) XRD plot of CDs. (b) TEM image of CDs, (c) particle size distribution plot of CDs, (d) HR-TEM images of CDs.

The crystalline character of CDs was determined by X-ray diffraction, which showed a broad peak at 22.28°, which corresponding to the (002) plane (Fig. 3a). The broad peak and the plane value directly represent the amorphous phase and irregular construction (arrangement) of the graphitic structure of the CDs. In addition, TEM and HR-TEM images revealed the structural features of CDs, which consisted of tiny spherical particles with an average particle diameter of 3.95 nm (Fig. 3b and c). Then, the *d*-spacing values of 0.35, 0.38, and 0.39 nm (Fig. 3d) further confirm the formation of a disordered graphitic-type structure. The disordered structure of CDs may be due to the presence of heteroatoms such as O and N, which modify the CD structure.

**Fig. 4:**
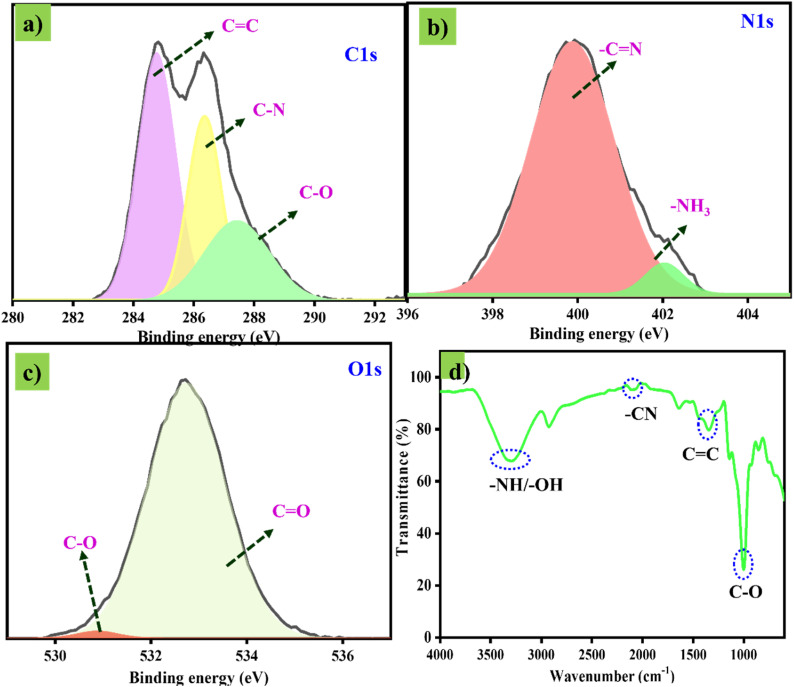
(a–c) Individual HR-XPS spectra of C 1s, N 1s and O 1s. (d) FT-IR spectrum of CDs.

Next, the principal surface analysis was examined by FT-IR to screen for functional moieties on the CDs. From the observed FT-IR outcomes (Fig. 4d), the CDs exhibit a broad peak at 3303 cm^−1^, indicating the presence of hydroxyl (–OH) and amine (–NH) groups with stretching vibrations. Then, a small peak at 2110 cm^−1^ confirms the presence of nitrile and carbonyl groups with stretching vibrations. Next, the peak at 1652 cm^−1^ indicates the presence of the C=C functional group,^20,21^ and the sharp peak at 1000 cm^−1^ represents the C–O functional group. From the spectral identification, FTIR confirms the presence of C=C and various functional groups in the CDs structure, indicating that the CDs have a uniform C=C core and well-arranged multiple functional moieties on their surfaces. Next, a thorough surface analysis of CDs was conducted using XPS, which provided deeper insights into the functional moieties and elemental composition of the CDs. The survey spectra (Fig. S1) of tartaric acid- and diamino propane-derived CDs show three major peaks located at binding energies of 284.5 eV, 389.3 eV, and 530.2 eV.

Furthermore, the C 1s spectra (Fig. 4a) were deconvoluted into three different peaks at 284.7, 286.37, and 287.4 eV, corresponding to the existence of C=C, C–N, and C–O/C–OH functional moieties. Then, the high-resolution N 1s spectrum (Fig. 4b), deconvoluted into two peaks at binding energies of 399.8 and 402.05 eV, indicates the presence of nitrogen-based functional groups, such as N–H and C–N–C. Similarly, the high-resolution O 1s spectrum (Fig. 4c) shows two distinct peaks at 530.8 and 532.72 eV, corresponding to C–O and C=O functional groups. From the observed three different HR-XPS spectra, it was clear that the formation of CDs is achieved by the contribution of C, N, and O-based moieties.^20,23,26^ The results from FT-IR and XPS confirm that CDs exhibit a core-surface-like structure.

### 3.3 Detection of environmental pollutants by CDs

**Fig. 5:**
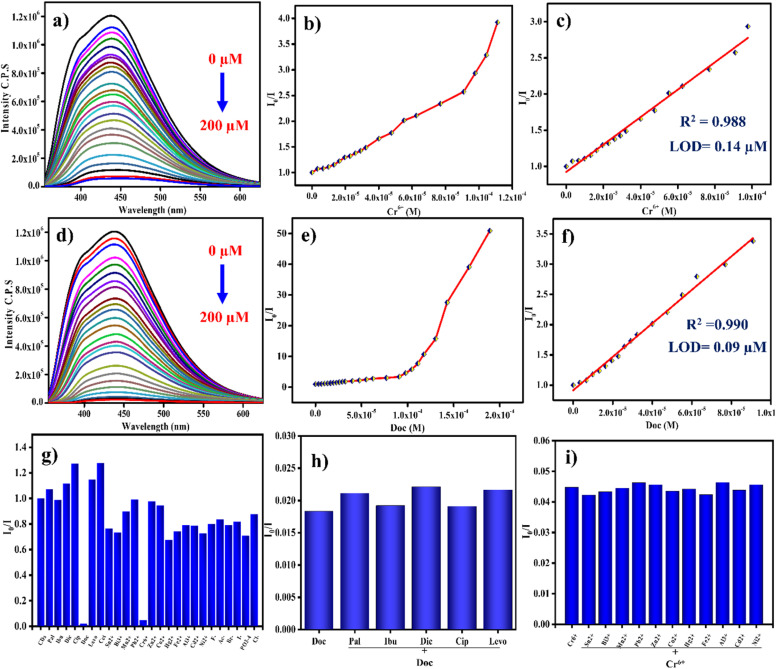
(a and d) CDs fluorescence quenching by Cr^6+^ and doxycycline. (b and e) response of fluorescence (*I*_0_/*I*) *vs.* varied concentration of Cr^6+^ and doxycycline. (c and f) Linear fit plot for CDs fluorescence quenching of (*I*_0_/*I*) *vs.* increasing concentration of Cr^6+^and doxycycline (Doc) respectively, (g) PL selectivity bar graph (*I*_0_/*I*) of CDs in the presence of different metal cations, anions and pharma products. (h) PL sensitivity of plot of CDs with Doc in the presence of other pharma compounds. (i) PL sensitivity of plot of CDs with Cr^6+^ in the presence of other metal cations and common anions.

The emission responses of as-prepared CDs and the listed toxic metal cations, common anions, and antibiotics were also tested using a photoluminescence-based fluorescence detection system in an aqueous medium. A general protocol for detecting toxins in aqueous conditions follows, recording the deviation in the CDs emission spectra in the presence and absence of toxic analytes at concentrations of 0–200 µM. The entire analytical mixture was immediately transferred to investigate the emission properties at 439 nm, excited at 350 nm. Each analyte was analysed individually, and the corresponding deviation in the fluorescence glow was recorded. Audition of diverse toxins such as heavy metal cations and a group of antibiotics with a concentration of exactly 200 µM in an aqueous environment, only Cr^6+^ metal cation and doxycycline (Doc) type antibiotic are the toxins that show a considerable variation in the noted PL spectrum, which was plotted as a bar graph (Fig. 5g). The variation in CDs PL intensity upon addition of the Cr^6+^ metal cation and (Doc) analytes reflects the detection capability of CDs.

Conversely, no other toxins affect PL nature of CDs. The above results confirm that CDs selectively detect the two different toxins. The Cr^6+^ ion and DOC selectively turn off the fluorescence of CDs without the involvement of any additional reagents. At a fixed emission wavelength of 439 nm, the emission intensity of CDs was progressively decreased with increasing concentration of analytes from (0–200 µM), and the quenching percentages of Cr^6+^ and Doc are 95 and 97% (Fig. 5a and d). Both contaminants exhibit excellent linearity in the recognition of specific analytes. A plot of linear relation among analyte concentration *vs.* PL emission intensity (*I*_0_/*I*) was obtained between the range of (0–100 µM) and (0–90 µM) with *R*^2^ values of 0.988 and 0.99 for Cr^6+^ and Doc, respectively (Fig. 5b and e). The observed deviation in fluorescence of the probe by adding analyte with increased concentration was fitted with the Stern–Volmer equation, and the Stern–Volmer quenching concentration was found as 1.8 × 10^4^ M^−1^ and 2.7 × 10^4^ M^−1^ for Cr^6+^ and Doc (Fig. 5c and f).^27^

1 *I*_0_/*I* = 1 + *K*_sv_[Q]

From the above formula, *I* and *I*_0_ describe the fluorescence intensity recorded at 439 nm and the presence and absence of Cr^6+^ or Doc with CDs. The next Q value represents the concentration of added analytes of Cr^6+^ or Doc. Next, using the linear fitted values and the formula 3*σ*/m, the lower limits of detection were calculated as 0.14 µM (140 nm) and 0.09 µM (90 nM) for Cr^6+^ and Doc, respectively. In Table S1 recent report on Cr and Doc detection *via* fluorescence was added. In continuation, the sensitivity of CDs with Cr^6+^ and Doc in the presence of other common cations, anions, and pharmaceutical compounds during the individual detection of water contaminants was investigated. Also, the sensitive of the CDs during the detection of different toxins was examined. The main objective of conducting the sensitivity experiment is to assess the tolerance ability of CDs when detecting the analyte in the presence of their own group materials (heavy metal ions and antibiotics) and anions. To ensure the probe's sensitivity to Cr^6+^ metal cations, 100 µM of interfering cations are added separately (Fig. 5i). Similarly, the selectivity of the CDs in the presence of Doc was also observed with other pharmaceutical pollutants (Fig. 5h). The obtained sensitivity outcome, in the presence of interferences, confirms that the CDs exhibit excellent selectivity and sensitivity in the specific recognition of water toxins.^28,29^

### 3.4 Mechanism for quenching of fluorescence by CDs

**Fig. 6:**
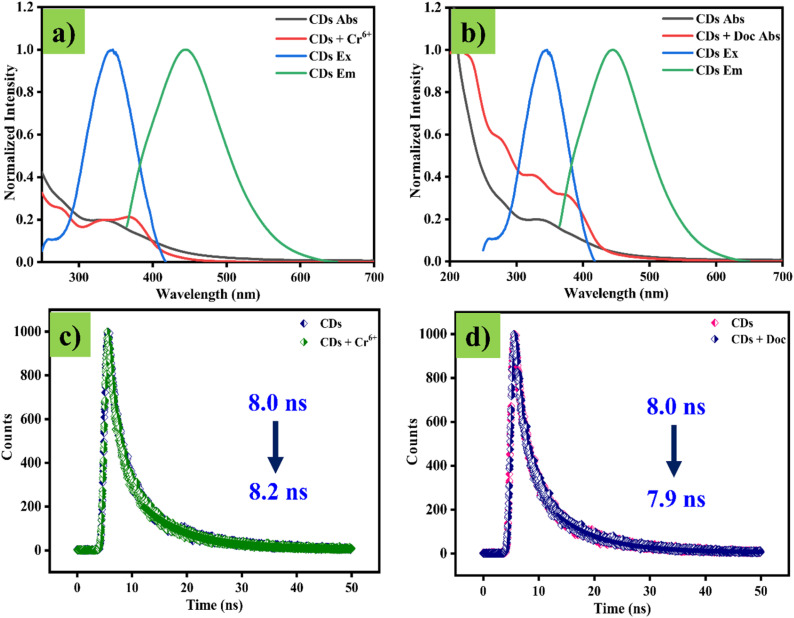
(a) Absorption of CDs with Cr^6+^ and excitation and emission of CDs. (b) Absorption of CDs with Doc and excitation and emission of CDs. (c) Average life time of CDs and CDs in presence of Cr^6+^. (d) Average life time of CDs and CDs in presence of Doc.

**Fig. 7:**
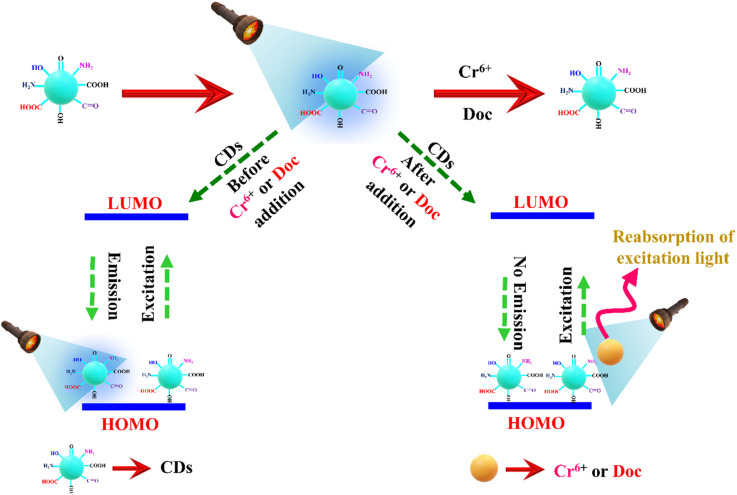
Proposed possible mechanism for the quenching (turn-off) fluorescence.

Various techniques, including UV, PL, and lifetime measurements, have been employed to determine the specific fluorescence-quenching mechanism of CDs in the presence of Cr^6+^ and Doc. We attempted to find the individual mechanism of fluorescence quenching by the Cr^6+^ and Doc. The common fluorescence quenching of emission in CDs is as follows, (1) inner filter effect (IFE) – represents the analyte's reabsorption of the excitation wavelength of the probe. (2) Photo-induced electron transfer (PET) corresponds to the transfer of electrons from the probe to an analyte in an excited state, (3) Förster resonance energy transfer mechanism (FRET) is detailed as the transfer of energy from a higher energy level donor to the lower energy level acceptor and (4) pH reduction leads turn off the CDs fluorescence by employment of Cr^6+^ and Doc.^28^ The quenching of fluorescence by the pH reduction mechanism was removed because, as the earlier discussion on pH stability clearly shows, the fluorescence intensity of the CDs increased under acidic environments. Similarly, the addition of Cr^6+^ and Doc to CDs increases the fluorescent intensity. Next, we investigated the possibility of an energy-transfer mechanism between the analytes and CDs. To achieve this, we examined the possibility of spectral overlap between the absorption spectra of CDs and individual analytes. However, the lack of spectral overlap between the analyte and the CDs' absorption spectra eliminates the FRET quenching mechanism. Furthermore, the absence of a wavelength shift in the CDs of the emission spectra with increasing analyte content supports the ruling out of the PET mechanism. To confirm the exact fluorescence turn-off mechanism of CDs by analytes such as Cr^6+^ and Doc. As mentioned earlier, absorption and excitation spectra of CDs with and without Cr^6+^ and Doc were recorded individually. In both cases of analyte addition, the absorption spectra of CDs with Cr^6+^ and Doc accurately overlapped with the excitation spectra of the CDs probe (Fig. 6a and b). The precise overlap of probe and analyte assures that turn-off of CDs emission occurred by only the inner filter effect IFE mechanism,^30^ which actually arose by reabsorption of the excitation light by the analyte instead of the probe, led to a reduction in the fluorescence emission nature of the CDs with increasing concentration of the Cr^6+^ and Doc.^31^ The quenching actually occurred due to the reabsorption of the excitation wavelength (light) by the analytes (Cr^6+^ and Doc) rather than by CDs, which is important for the reduction in the emission character of the CDs. Due to insufficient excitation energy, the fluorescence of the CDs was completely quenched as the analyte concentration increased. Next, it was well known that the quenching CDs was occurred either by static and/or dynamic processes based on the interaction and modification in the average lifetime of the probe by analyte; before that, the calculated larger *k*_sv_ of 1.8 × 10^4^ M^−1^ and 2.7 × 10^4^ M^−1^ for Cr^6+^ and Doc suggested that the process of CDs fluorescence quenching by the (Cr^6+^ and Doc) is purely based on either static or dynamic process, surely not an combination of both static and dynamic process.^32,33^ Furthermore, the exact quenching process was confirmed by measuring the average lifetime, which was calculated as 8.0 ns for the probe (CDs), and with analytes Cr^6+^ and Doc, calculated individually as 8.2 ns and 7.9 ns (Fig. 6c and d). Almost the entire lifetime was unaltered by adding analytes, suggesting that a non-fluorescent complex formed, which led to a static quenching process. The mechanism of CDs fluorescence quenching by the Cr^6+^ and Doc was cantonized in the (Fig. 7).

### 3.5 CDs for security applications

**Fig. 8:**
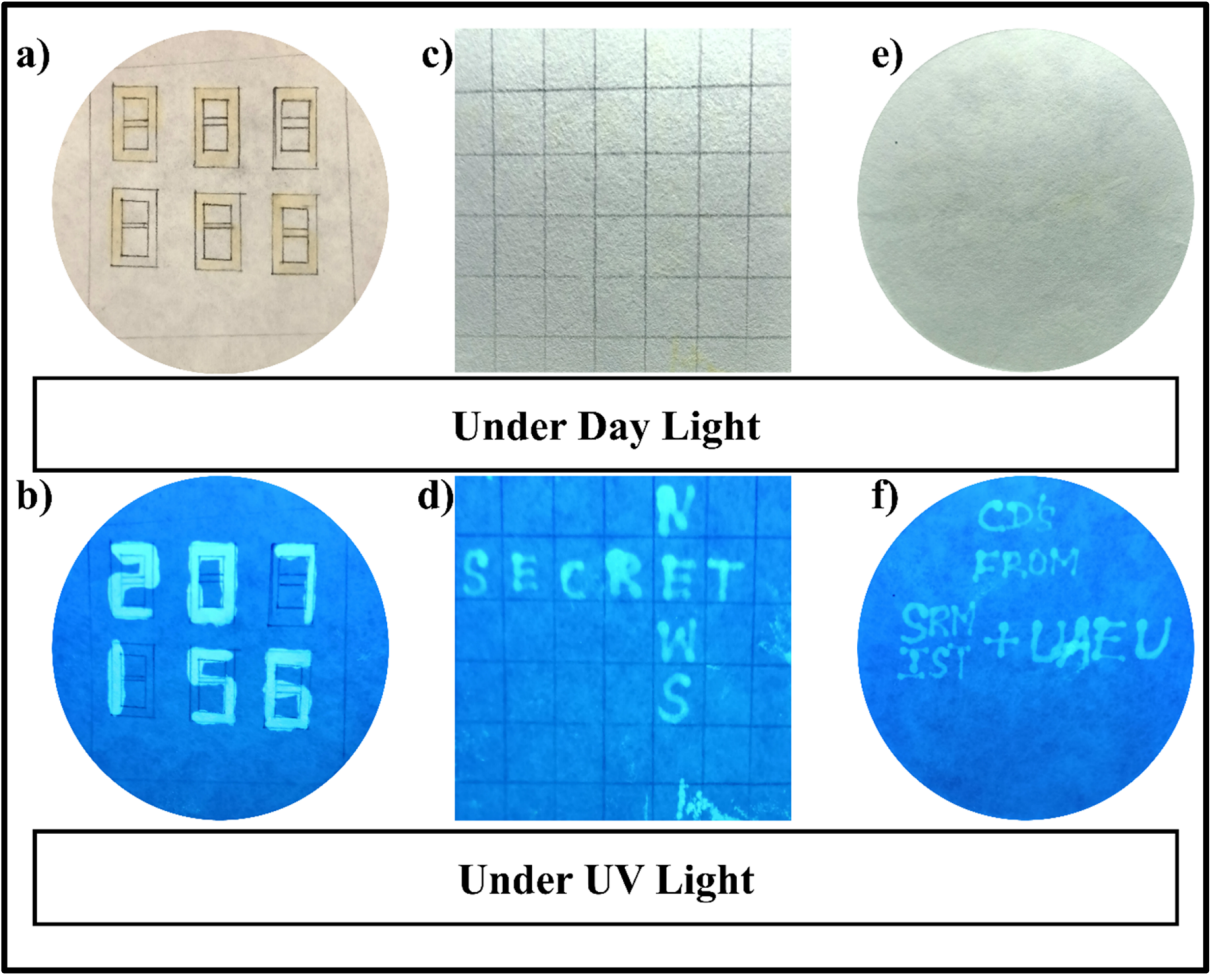
(a and b) Secret number written in filter paper with CDs under visible light and UV light, (c and d) secret words written in filter paper with CDs under visible light and UV light, (e and f) fluorescent ink application of CDs under visible and UV light.

Now, information technology has grown immensely globally, and various advanced techniques have been developed and are available for transporting confidential information. The hacking technology also developed simultaneously, enabling it to steal critical information and create issues easily. For that anticounterfeiting information, transportation will be recommended, as it uses fluorescent materials that are invisible to the naked eye in daylight but clearly visible under UV light. Anticounterfeiting methods can be easily transferred without the leakage of confidential information, particularly within the department of defence. Various conventional fluorescent dyes are employed as fluorescent inks, including stilbene, Coumarin, and anthracene. However, the major issue with those dyes is that they are highly carcinogenic to the ecosystem, costly, and lose their fluorescent properties with prolonged exposure to light. At this time, CDs have a strong capacity to substitute for conventional fluorescent dyes because they possess excellent water solubility, exhibit strong fluorescence with excitation-dependent behaviour, remain stable under continuous light irradiation, and are comparatively lower in cost.^34^ Here, we have used the CDs prepared from simple precursors like tartaric acid and diaminopropane were additionally evaluated to unknot their potential in fluorescent ink characteristics focused on anticounterfeiting and secured information transportation. The CDs already have very good water solubility, which facilitates uniform dispersion and a simplest way to employ as ecofriendly aqueous printing ink, interestingly does not require any additional protecting agents to maintains its stability like conventional quantum dots. Under normal condition and light, the paper written with CDs ink remained invisible, but there will be an intense blue emission was observed when UV light touches it, confirms the capability of secret making (Fig. 8a). shows the secret numbers written in the rectangular 888 box under visible light, and the secret numbers are visible under the UV light illumination (Fig. 8b). In (Fig. 8c), a word was written in a puzzle-like sheet, but it looked empty under visible light, and there was no evidence for any written words on it. After exposing it to UV light, the word is visible as “SECRET NEWS”. Similarly (Fig. 8e and f), shows the words CDs from SRM IST and UAEU under UV and Visible light irradiation. The strong and stable emission nature of the CDs provides an long term security protection, which reduces the costs from further replacement. The aqueous CDs ink posses very good photostability and maintains its intensity for prolonged light exposure makes it a sustainable and stable material for several days. These features of CDs as luminescent inks for anticounterfeiting and confidential news hiding advanced in defence area.

## 4 Conclusion

In this work, we have presented and discussed CDs with multiplex characteristics prepared using simple precursors and a straightforward, rapid method. The as-prepared CDs are subjected to comprehensive characterization confirms that the CDs consist of a uniform core coupled with the electron rich functional groups and offer excellent optical properties under multiple environmental conditions. The outstanding fluorescent characteristics of the CDs enable dual detection of carcinogenic Cr^6+^ and doxycycline individually in aqueous conditions, with comparable detection limits of 140 nM and 90 nM, respectively. The detection capability of CDs maintained excellent selectivity and sensitivity even in complex environments. Beyond environmental pollutant sensing, the CDs exhibit an interesting practical potential as fluorescent inks for safe and secure information encryption and anti-counterfeiting applications under UV illumination. These results highlight the wide range of roles and applications of CDs as low-cost fluorescent materials in environmental monitoring and information security. The multiplex nature of the CDs paves the way for developing a sustainable platform for water quality monitoring and highly secured communication technologies.

## Conflicts of interest

There are no conflicts to declare.

## Supplementary Material

RA-016-D5RA08943C-s001

## Data Availability

Data will be made available on request. Supplementary information (SI) is available. See DOI: https://doi.org/10.1039/d5ra08943c.
