# Supplementary material for: Bottom-up microwave transformation of molecules to carbon dots for detection and encryption applications
Source: RSC Adv. 2026 Jan 21;16(5):4618–28. doi: 10.1039/d5ra08943c (PMC12821142; doi:10.1039/d5ra08943c)
Supplement: RA-016-D5RA08943C-s001 [file RA-016-D5RA08943C-s001.pdf]

Supplementary Information (SI) for RSC Advances.
This journal is © The Royal Society of Chemistry 2026

Bottom-up Microwave Transformation of Molecules to Carbon Dots for Detection and Encryption Applications

Arun Annamalai[a,b], Sundaravadivel Elumalai[c*], Sambasivam Sangaraju[b*], Fathy M. Hassan[a,b*]

a. Department of Chemistry, United Arab Emirates University, Al Ain - 15551, UAE.
b. National Water and Energy Center, United Arab Emirates University, Al Ain 15551, UAE.
c. HIDE- Laboratory, Department of Chemistry, Faculty of Engineering and Technology, SRM Institute of Science and Technology, Kattankulathur, Tamil Nadu, 603203, India.

* Corresponding author's E-mail:
sundaravadivelchem@gmail.com ; s_sambasivam@uaeu.ac.ae; f.hassan@uaeu.ac.ae

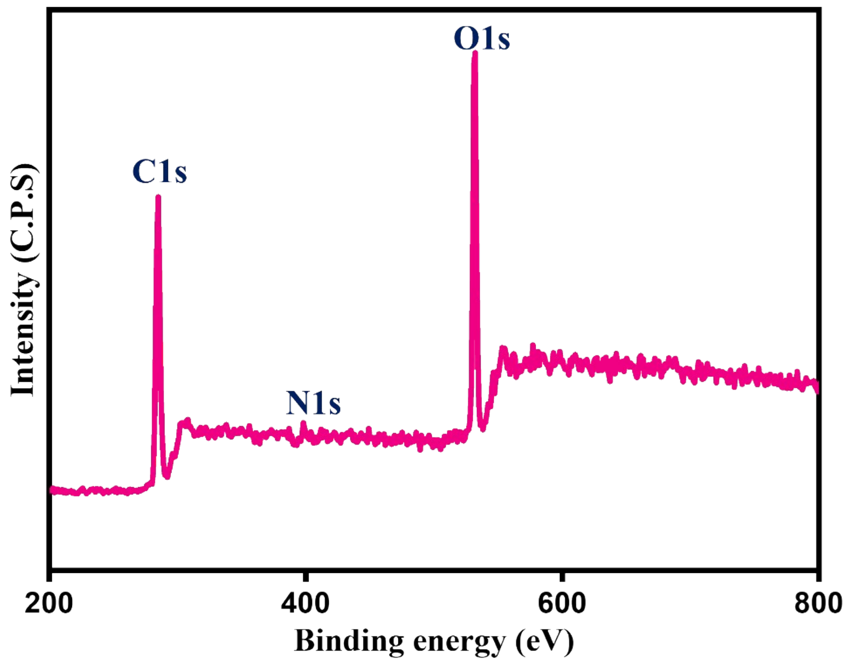

Figure S1: Survey spectra of CDs.

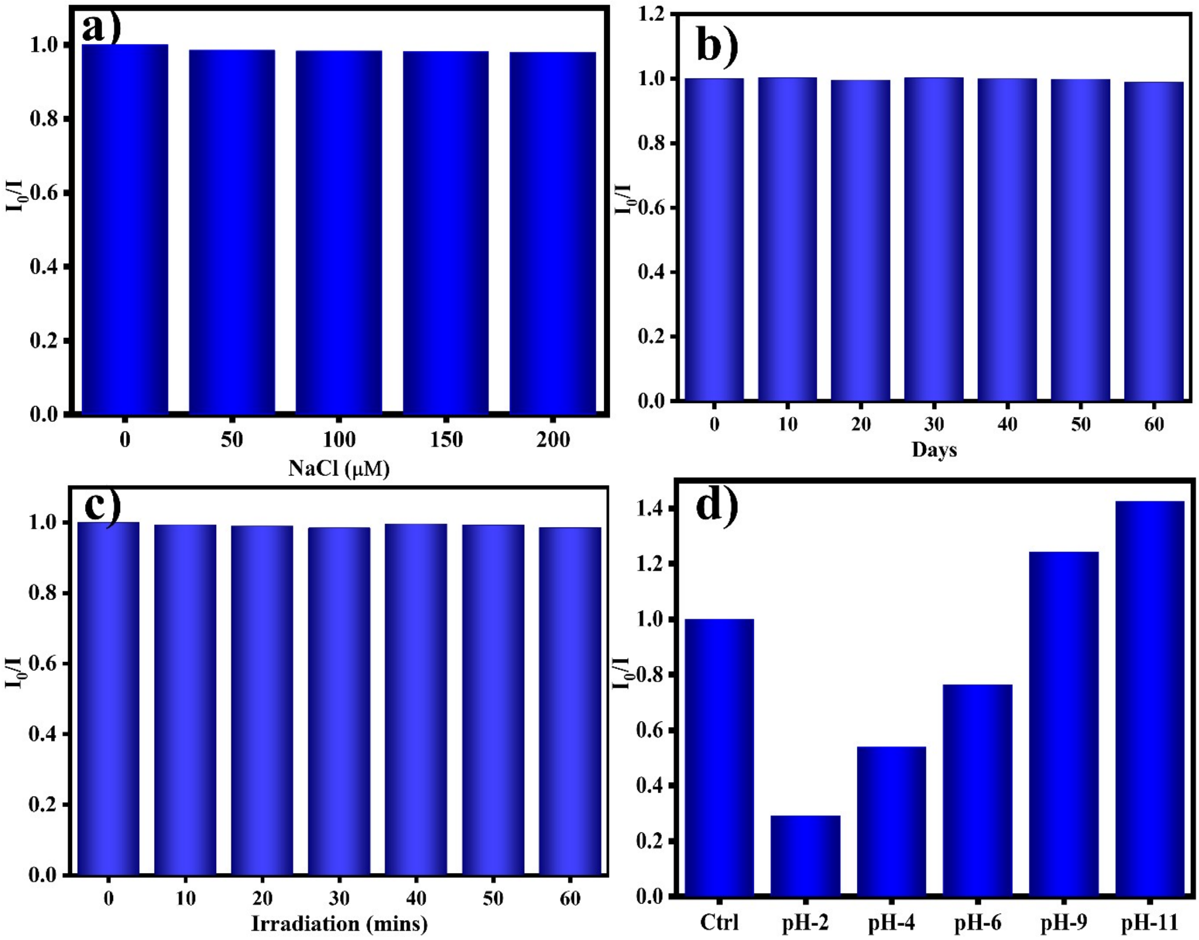

Figure S2: Stability of CDs under a) Ionic environment, b) Long duration of storage c) UV-light irradiation, d) Different pH environment.

Table S1:

Table for comparing the present work LOD of Cr^{6+} and Doc with already reported values based on fluorescence-based sensor probes.

Detection probe	Contaminant detected	Linear range	Detection Limit	Reference
**CDs**	**Cr^{6+}**	**0-100 µM**	**0.14 µM (140 nM)**	**This Work**
N-CDs	Cr^{6+}	1-100 µM	0.12 µM	[1]
CDs@Eu-MOFs	Cr^{6+}	2-100 µM	0.21 µM	[2]
N, S-CDs	Cr^{6+}	1-10 µM	0.2 µM	[3]
CDs-Ws	Cr^{6+}	0-50 µM	106.57 nM	[4]
CDs	Cr^{6+}	3-50 µM	0.09µM	[5]
Ru@CDs	Cr^{6+}	0-150 µM	0.128 µM	[6]
CDs	Cr^{6+}	0-117 µM	0.11 µM	[7]
CDs	Doc	10-1000 µM	16.35 µM	[8]
$CDs@CaF_2$: Eu^{3+}	Doc	0.1-30 nM	43 nM	[9]
S-Dots	Doc	0.5-25 µM	0.19 µM	[10]
CDs	Doc	0.05-500 µM	18 nM	[11]
S,N-CDs	Doc	0.5-500 µM	25 nM	[12]
**CDs**	**Doc**	**0-90 µM**	**0.09 µM (90 nM)**	**This work**

References

[1] M. Cao, Y. Li, Y. Zhao, C. Shen, H. Zhang, and Y. Huang, "A novel method for the preparation of solvent-free, microwave-assisted and nitrogen-doped carbon dots as fluorescent probes for chromium(VI) detection and bioimaging," *RSC Adv*, vol. 9, no. 15, pp. 8230–8238, Mar. 2019, doi: 10.1039/C9RA00290A.

[2] Y. Wang, J. He, M. Zheng, M. Qin, and W. Wei, "Dual-emission of Eu based metal-organic frameworks hybrids with carbon dots for ratiometric fluorescent detection of Cr(VI)," *Talanta*, vol. 191, pp. 519–525, Jan. 2019, doi: 10.1016/J.TALANTA.2018.08.078.

[3] H. Zhang, Y. Huang, Z. Hu, C. Tong, Z. Zhang, and S. Hu, "Carbon dots codoped with nitrogen and sulfur are viable fluorescent probes for chromium(VI)," *Microchimica Acta*, vol. 184, no. 5, pp. 1547–1553, May 2017, doi: 10.1007/S00604-017-2132-4/TABLES/2.

[4] X. Ji *et al.*, "Green synthesis of weissella-derived fluorescence carbon dots for microbial staining, cell imaging and dual sensing of vitamin B12 and hexavalent chromium," *Dyes and Pigments*, vol. 184, p. 108818, Jan. 2021, doi: 10.1016/J.DYEPIG.2020.108818.

[5] A. Annamalai, K. Annamalai, R. Ravichandran, S. Bharathkumar, and S. Elumalai, "Multi-functional carbon dots from simple precursors: An excellent heavy metal ions sensor with photocatalytic activity in aqueous environment," *Colloids Surf A Physicochem Eng Asp*, vol. 652, Nov. 2022, doi: 10.1016/j.colsurfa.2022.129800.

[6] A. Annamalai, K. Annamalai, R. Ravichandran, A. K. Anilkumar, G. M R, and S. Elumalai, "Simple synthesis of water-soluble ruthenium carry CDs as admirable probe for improved environment and biological applications," *Mater Today Chem*, vol. 30, Jun. 2023, doi: 10.1016/j.mtchem.2023.101536.

[7] K. Annamalai, A. Annamalai, R. Ravichandran, and S. Elumalai, "Recyclable waste Dry-cell batteries derived carbon dots (CDs) for detection of Two-fold metal ions and degradation of BTB dye," *Waste Management*, vol. 163, pp. 61–72, May 2023, doi: 10.1016/j.wasman.2023.03.032.

[8] Q. Su, X. Wei, J. Mao, and X. Yang, "Carbon nanopowder directed synthesis of carbon dots for sensing multiple targets," *Colloids Surf A Physicochem Eng Asp*, vol. 562, pp. 86–92, Feb. 2019, doi: 10.1016/j.colsurfa.2018.11.015.

[9] X. Tian and Z. Fan, "Novel ratiometric probe based on the use of rare earth-carbon dots nanocomposite for the visual determination of doxycycline," *Spectrochim Acta A Mol Biomol Spectrosc*, vol. 260, Nov. 2021, doi: 10.1016/j.saa.2021.119925.

[10] Y. Zhuang *et al.*, "A ratiometric fluorescent probe based on sulfur quantum dots and calcium ion for sensitive and visual detection of doxycycline in food," *Food Chem*, vol. 356, Sep. 2021, doi: 10.1016/j.foodchem.2021.129720.

[11] Y. Feng, L. Tan, Q. Tang, W. Zhong, and X. Yang, "Synthesis of Carbon Dots from PEG6000 and Papain for Fluorescent and Doxycycline Sensing," *Nano*, vol. 13, no. 9, Sep. 2018, doi: 10.1142/S1793292018501060.

[12] J. Song *et al.*, "A novel fluorescent sensor based on sulfur and nitrogen co-doped carbon dots with excellent stability for selective detection of doxycycline in raw milk," *RSC Adv*, vol. 7, no. 21, pp. 12827–12834, 2017, doi: 10.1039/c7ra01074e.
